# The impact of Tai Chi and mind-body breathing in COPD: Insights from a qualitative sub-study of a randomized controlled trial

**DOI:** 10.1371/journal.pone.0249263

**Published:** 2021-04-08

**Authors:** Elizabeth A. Gilliam, Tina Cheung, Kristen Kraemer, Daniel Litrownik, Peter M. Wayne, Marilyn L. Moy, Gloria Y. Yeh

**Affiliations:** 1 Division of General Medicine, Department of Medicine, Beth Israel Deaconess Medical Center, Brookline, MA, United States of America; 2 Harvard Medical School, Boston, MA, United States of America; 3 Osher Center for Integrative Medicine, Harvard Medical School and Brigham and Women’s Hospital, Boston, MA, United States of America; 4 Pulmonary and Critical Care Medicine Section, Department of Medicine, Veterans Administration Boston Healthcare System, Boston, MA, United States of America; The MetroHealth System and Case Western Reserve University, UNITED STATES

## Abstract

**Purpose:**

Chronic obstructive pulmonary disease (COPD) is associated with multiple psychosocial and behavioral factors. Prior research suggests that mind-body interventions may support the development and maintenance of healthy behaviors and improve health-related quality-of-life in such patients. We sought to qualitatively explore cognitive, psychosocial, and behavioral changes in patients with COPD who participated in two different mind-body interventions compared to an education control.

**Methods:**

We analyzed semi-structured qualitative exit interviews from a prospective, randomized pilot trial (N = 123) investigating 12-weeks of Tai Chi (TC) vs. mind-body breathing (MBB) vs. education (EDU) control in patients with moderate-severe COPD. TC involved traditional movements, that integrate meditative breathing, while MBB focused mainly on meditative breathing techniques alone. Interviews were audio-recorded and transcribed verbatim. Qualitative analysis of randomly selected transcripts was performed by two independent reviewers using an iterative process to identify emergent themes informed by grounded theory methods until thematic saturation was reached.

**Results:**

A total of 66 transcripts were reviewed (N = 22 TC, N = 22 MBB, N = 22 EDU). Participants were mean age = 68.1 years, GOLD Stage = 2.3, baseline FEV1_1_ percent predicted mean (SD): 58% (13.4), 42.4% female. We identified six frequently mentioned themes: 1) overall awareness and understanding, 2) self-care knowledge, skills and behaviors, 3) behavior-related neurocognitive concepts, 4) physical function, 5) psychological well-being, and 6) social support/social function. Compared to EDU, more participants in TC and MBB noted improvements in awareness of self and the mind-body connection (e.g., body and breath awareness), knowledge of breathing techniques and integration of self-care skills with daily activities, self-efficacy for symptom management (particularly managing anxiety and dyspnea), acceptance of disease, physical function improvements (e.g., endurance, dyspnea, fatigue), and psychological well-being (particularly relaxation, emotion regulation and decreased reactivity). Compared to MBB, those in TC shared more intention to continue with self-care behaviors, physical activity self-efficacy, and improved flexibility. All three groups, including EDU, noted increased social support and knowledge of disease. Those in EDU, however, had fewer mentions of processes related to behavior change, and less concrete changes in neurocognitive, psychological, and physical function domains.

**Conclusions:**

Mind-body interventions including meditative breathing may impact behavior-related neurocognitive and emotional factors that improve self-care management and support positive behavioral changes in patients with COPD.

**Trial registration:**

This trial is registered in Clinical Trials.gov, ID number NCT01551953.

## Introduction

Chronic obstructive pulmonary disease (COPD) is a syndrome of progressive airflow limitation [1] resulting in breathlessness and limitations in physical function [1, 2] and is a major cause of morbidity and mortality both in the US and worldwide. As COPD is a complex chronic illness, the importance of the biopsychosocial model is increasingly recognized, and current management guidelines emphasize a multimodal approach that holistically addresses the patient experience. It is well-known that psychosocial factors such as anxiety, depression, fatigue, and social isolation have an important role in clinical functioning and well-being [3–8]. Prior research suggests that mind-body interventions may support the development and maintenance of healthy behaviors and improve health related quality of life (HRQL).

Integrative mind-body programs, such as Tai Chi, have been explored for COPD management as they may impact relevant physiological and psychosocial pathways. Tai Chi incorporates aerobic, strength, and balance training with breathing techniques, mindfulness, and focused internal awareness [9–11]. Previous research [12–16] has suggested that Tai Chi and similar mind-body behavioral interventions may have beneficial effects in COPD patients on HRQL, symptoms of anxiety and depression, and physiological measures like pulmonary function and exercise capacity [17–21].

There has also been a growing literature supporting mind-body interventions and positive behavior change. Mind-body interventions, including Tai Chi, have been shown to improve key processes implicated in health behavior change, such as self-efficacy, emotion regulation, and executive functioning [22–24]. Improvements in these processes may, in turn, lead to downstream improvements in health behavior change [25]. Indeed, although there is limited work in Tai Chi specifically, extant work suggests that mind-body interventions (e.g., mindfulness interventions) may improve healthy behaviors, such as physical activity, smoking cessation, and adherence to medical regimens [26].

One component of Tai Chi with particular relevance for the COPD population is mind-body breathing. Mind-body breathing strategies foster an interoceptive [27] awareness of somatic and psychological processes associated with breath, and provide a tool for focused mindful attention [28, 29]. Often, mind-body breathing can also include a purposeful slow, deep breathing that encourages utilization of full lung capacity and complete exhalation. Slow breathing patterns can positively impact both physiological and psychological health outcomes, including heart rate variability, blood pressure, mood, and disease related quality of life [30–33] in both COPD and non-COPD populations [34]. The extent to which mind-body breathing is beneficial, in isolation from the multimodal Tai Chi intervention, is not well understood. Similarly, the impact of Tai Chi and mind-body breathing on behavior change is understudied.

Mixed method approaches that utilize qualitative in addition to traditional quantitative analysis are increasingly recognized as valuable for exploring the complexities of patient experiences and behaviors and contextualizing quantitative outcomes [35, 36]. Qualitative research, which can descriptively describe the experiences of a sample and capture themes not found by quantitative instruments, are often used to compliment and inform quantitative results and generate hypotheses. We conducted a pilot [37] feasibility randomized controlled trial examining the effects of Tai Chi and a mind-body breathing intervention (adapted from the Tai Chi protocol) on exercise capacity and HRQL in individuals with COPD. In the context of this trial, we sought to qualitatively characterize patient experiences, better understand emergent patient-centered outcomes, and explore behavioral changes in the two intervention groups compared to an education control group.

## Methods

### Quantitative parent study

The parent study [37] (trial registration number NCT01551953) was a prospective, randomized controlled trial investigating 12-weeks of Tai Chi (TC) vs. education (EDU) control vs. a third exploratory arm of mind-body breathing (MBB). This study was approved by the Beth Israel Deaconess Medical Center Institutional Review Board (protocol #1020P-000412). Written informed consent was obtained from all participants. In brief, we randomized 123 participants with moderate to severe COPD recruited from outpatient academic medical clinics in the Boston area in a 2:1:1 ratio to 12 weeks of twice weekly TC (N = 61), EDU (N = 31), or MBB (N = 31). See S1 Appendix for the Consort Flow Diagram. We included participants with moderate-severe COPD as defined by (1) GOLD (Global Obstructive Lung Disease) stage 2 or 3 or 4 with symptoms of dyspnea (either FEV_1_ ≤ 80% and FEV_1_/FVC <0.70, or CT evidence of emphysema) and (2) age ≥ 40 years. We excluded those with: 1) respiratory failure or GOLD stage 4 who were unable to perform a 6 minute walk test (6MWT); 2) COPD exacerbation requiring steroids, antibiotics, ED visit or hospitalization within the past 2 weeks; 3) planned thoracic surgery within the next 3 months; 4) hypoxemia on 6MWT or cardiopulmonary exercise test (oxygen saturation < 88% on supplemental oxygen); 5) inability to ambulate due to vascular or other neuromuscular conditions that would preclude a 6MWT; 6) clinical signs of unstable cardiovascular disease (i.e., chest pain on walk test or EKG changes on cardiopulmonary exercise test); 7) severe cognitive dysfunction (Mini-Mental Status Exam ≤ 24); 8) non-English speaking; 9) current active participation in pulmonary rehabilitation program or current regular practice of Tai Chi. The first study enrollment was 8/29/2011 and the last data collection date was 3/2/2016.

The TC intervention, previously designed for an older, physically limited population, consisted of five Tai Chi movements based on the traditional Cheng Man-Ch’ing’s Yang-style short form [9, 38, 39]. Four breathing techniques were also integrated: 1) “Renewing the body with breath”, which emphasizes relaxation, body and breath awareness, and imagery to systematically scan the whole body and release tension; 2) “Mindful Breathing,” which emphasizes mental focus, interoception, awareness of the mechanics of breathing; 3) “Dan Tien Breathing” or “Ocean Breathing”, which combines diaphragmatic breathing with arm movements and mental imagery; 4) 4) “Balloon Breathing,” which extends the previous practice by extending the period of exhalation. Each class began with a series of warm-ups.

The MBB intervention was designed to emphasize the mind-body breathing component of the TC group but exclude the traditional movements. The same five breathing techniques as taught in TC made up the main portion of each MBB class. Practice of the breathing techniques was integrated into simulated activities of daily living (e.g., walking, folding laundry, washing dishes).

The education EDU control was designed to replicate the social interaction of the intervention arms, but without any physical activity, breathing exercise, or mind-body components. Classes for this group focused on the presentation and discussion of educational material from the American College of Chest Physicians, the American Thoracic Society, and the Global Obstructive Lung Disease Patient Guide. Educational modules included: anatomy of the lungs, COPD, managing COPD symptoms, smoking cessation, diagnostic tests, understanding COPD meds, managing acute exacerbations, managing stress, exercise, nutrition, sleep, mental health, oxygen therapy, surgical options, pulmonary rehabilitation, and advance care planning. Time was spent with both didactic as well as informal group discussion moderated by the instructor.

This education content (printed slides) was also provided to patients in TC and MBB groups, although not presented and discussed as in EDU.

### Qualitative sub-study

Semi-structured interviews were conducted with all participants at the end of the 12-week intervention. Open ended questions focused on the participant’s experience with the intervention and any perceived changes in physical and mental wellbeing or functioning as a result of the study (S2 Appendix). The same study member (DL), who was experienced with qualitative methods, performed all interviews. Interviews were audio recorded and transcribed verbatim. Our goal in analyzing these transcripts was to provide qualitative insight and generate hypotheses regarding pathways by which mind-body therapies may impact people with COPD.

Qualitative analysis was performed on a randomly selected subset of transcripts from each study group by two independent reviewers (EG, TC) using an iterative process to identify and explore emergent themes informed by grounded theory methods [40]. An initial sample of 15 transcripts, 5 from each of the three study groups, was analyzed and discussed among the authors, leading to the development of a preliminary list of themes and subthemes. This list was expanded and revised when coding subsequent transcripts, with analysis continuing in batches of 5–6 per group to the point of thematic saturation [40].

Extracted information included broad themes that were identified de novo, nuanced details or sub-themes that emerged, and relevant, representative quotes pertaining to each theme. The number of participants within each group who mentioned a given theme during their interview was also noted. Categories were not meant to be completely mutually exclusive. When a specific statement fit into more than one category, it was assigned by consensus to the single theme it most specifically represented. A single quote could contain multiple statements. Categorization of themes, and further synthesis and analysis proceeded through multiple collaborative discussions.

We then qualitatively described, compared, and contrasted the groups with respect to the occurrence of themes and number of participants endorsing each theme. Bias and validity were addressed through random selection of transcripts for analysis, having each transcript independently coded by two authors who had not taken part in the original interviews, having a third author (GY) act as arbiter in cases where there was disagreement, utilizing a multi-disciplinary analysis team, and documenting all analysis and decision points in detail.

## Results

A total of 66 qualitative transcripts, 22 from each group, underwent review, coding, and analysis before thematic saturation was reached. The qualitative sample represented 76% of the total transcripts from the parent study. Baseline characteristics of this sub-population (N = 66) included 42.4% female, mean age = 68.1 years, GOLD Stage = 2.3, baseline FEV_1_ percent predicted = 58% and were generally representative of the overall study population. Additional demographic data of the total sample is published elsewhere [41].

Six broad themes emerged from the qualitative interviews: 1) overall awareness and understanding, 2) self-care knowledge, skills, and behaviors, 3) behavior-related neurocognitive concepts, 4) physical function, 5) psychological wellbeing, and 6) social support/social function. Within each domain, several related themes or sub-themes emerged. To aid in qualitative comparison Table 1 shows the percentage of individuals within each group who endorsed a given theme at least once during their interview. Boxes 1–6 provide representative quotes from each of the main domains.

**Table 1:** Percent of individuals per group endorsing emergent themes.

**OVERALL AWARENESS and UNDERSTANDING**	**TC %**	**EDU %**	**MBB %**
Of Self	73	5	77
Of Disease (COPD)	23	45	41
Of Mind-Body Connection	32	0	45
**SELF-CARE KNOWLEDGE, SKILLS, and BEHAVIORS**	**TC %**	**EDU %**	**MBB %**
Knowledge of Breathing Techniques	55	23	82
Knowledge of Self Care Strategies	64	50	77
Skills Integration into Daily Life	86	27	86
Physical Activity Engagement	32	27	23
Engagement in Other Healthy Behaviors (Smoking Cessation, Diet, Medication Adherence)	18	9	27
**BEHAVIOR-RELATED NEUROCOGNITIVE CONCEPTS**	**TC %**	**EDU %**	**MBB %**
Internal Locus of Control for Disease Management	36	9	64
Gaining a New Tool or Resource	27	0	32
Self-Efficacy for Symptom Management	45	14	50
Self-Efficacy for Managing Anxiety and Breathlessness	27	5	18
Self-Efficacy for Physical Activity	23	5	9
Acceptance of Disease	45	9	32
Intention to Continue Self Care	50	5	9
**PHYSICAL FUNCTION**	**TC %**	**EDU %**	**MBB %**
Improved Physical Functioning	55	5	59
Reduced Dyspnea	36	9	32
Reduced Fatigue / Improved Energy	45	0	36
Reduced Need for Rescue Medications	32	9	14
Improved Flexibility	27	0	5
**PSYCHOLOGICAL WELLBEING**	**TC %**	**EDU %**	**MBB %**
Relaxation	86	0	82
Emotional Regulation	59	0	64
Appreciation	23	5	9
Less Emotional Distress	45	9	50
Improved Outlook	32	5	50
**SOCIAL SUPPORT / SOCIAL FUNCTION**	**TC %**	**EDU %**	**MBB %**
Camaraderie	64	45	41
Seeing Others With the Same Disease	55	36	27
Shared Difficult Experience	41	23	36
Sharing Knowledge with Other Patients	14	41	27
Improved Social Engagement or Increased Social Role	32	14	23

## Box 1. Representative quotes related to ‘overall awareness and understanding’

**Table:** 

**Of Self** • “Just making yourself kind of slow down and…just being aware of what’s going on and your breathing…I never thought about my breathing until I went home from these classes. “(TC) • “I’m more in tune with my body and the areas that hurt. And it’s not a negative thing. It’s where I can actually work through the pain. . .I’m more in tune with what hurts and what to do in order to not make it hurt more.” (TC) • “I never thought about the mental side of it, I just thought I had a couple of crappy lungs and there’s nothing really you can do about it…putting yourself in the right mental state, helps you so much more.” (TC) • “[The classes] made me more mindful…[I] check in with my breathing periodically during the day, which I never did… I’m aware of it. And I’m breathing better and I’m breathing deeper.” (MBB) **Of Disease** • “I know more about it now. I can say chronic obstructive pulmonary disease. I’ve got it all memorized now.” (TC) • "I understand better now…as far as your lungs, your heart, your blood and everything, and how it’s incorporated. And now I realize this because the booklet, we’re reading through it and understanding lung function and body function–that’s helped me a lot." (MBB) • "I’ve never really understood the disease. I only had the disease…but when you’re in a class with everybody that has the disease, and they’re talking about it, that’s the educational component I was really interested in." (MBB) • “Any time that you’re forced to look at something in a different manner…it broadens your understanding of that situation and that’s certainly the case with my emphysema…I know a lot more now” (MBB) **Of Mind-Body Connection** • “These things affect you mentally, emotionally, physically. It’s all joined in together, so when you’re stressed, and you’re angry, or somebody hurt your feelings, or things don’t go right on a job, or you’re family having a fight…it affects your breathing because you’re not breathing according to how your emotions are carrying you and that makes a difference.” (TC) • “It’s mind-body. It’s not just body. When I look at exercise, there’s a connection…like runners, they get off their high. But the connection is not the same, or as in-depth, as doing the Tai Chi. . .and the overall mental and physical benefits (with Tai Chi).” (TC) • “The gym is you gotta lift the weights, and do a gazillion treadmills…but this is more psychological, and it works better for your body…, I feel much better now, because I’m thinking about what’s going on…how I’m breathing. I’m thinking of my whole body now…It gives you a better focus.” (TC) • “I’ve learned now, your mind actually controls your whole body. And when you can control your breathing…your nerves will quiet down and you’ll get more oxygen and you’ll be more restful.” (MBB)

## Box 2. Representative quotes related to specific self-care knowledge, skills, and behaviors

**Table:** 

**Knowledge of Breathing Techniques and of Self Care Strategies** • “[You learned]…how to channel your inner ability to breathe in a way that’s…helpful for you…I know for a fact that even if you have asthma, COPD, any kind of lung problem, it’s important to stay active, physically, you got to keep it moving. . .you may not exercise to a point a younger person does, or go as fast, but the thing is to stay active, keep it moving, because if you do that it’s better for your joints, it’s better for your lungs,." (TC) • “My breathing is totally different now than it was before…[Tai Chi] is just keeping the rhythm, and the momentum, and feeling everything…I’ve learned you have to get the oxygen out of your lungs…you gotta inhale and exhale, empty your lung[s].” (TC) • “It does make me more aware of either trying not to get myself into a situation where I’m going to have a problem…[or] saying, okay you can do this, you just have to do it with a different pace.” (MBB) • “That hadn’t really occurred to me before, that I could do things slowly, I always tried to keep up with everybody else, but I walk at the pace that I thought was normal and I realize that I can control my oxygen, just walking a little more slowly than I would normally.” (EDU) **Skills Integration into Daily Life** • “When I went to surgery…I’m moving my foot (in a Tai Chi movement) to keep me calm. . .Even when I sit down in my living room, I go like this (Tai Chi breathing). . .I find that what I learned in some of the classes, I’m doing it. I’m doing it now in everyday life.” (TC) • “It has taught me how to go up those steps without stopping, I used to stop before I get to the top…it has taught me if I breathe right, I can go from step to the last step, and then I relax when I get to the top.” (MBB) • “Instead of doing the exercise being totally out of breath, you do the exercise at a nice pace and do the right breathing techniques. . .you catch yourself not doing it at first…you think about what you learned in class and you adjust yourself…if I do situps now, I’m breathing. . .I’m not out of breath when I’m done…it feels better. (MBB) • "I can give you a hundred places where I’d do [what I learned in class]. In the dining room. . .When I walk around with my wife in the supermarkets…when I’m brushing my teeth. . .then when I get in the shower, I’m doing something." (MBB) **Physical Activity Engagement** • “When I joined the study I wasn’t doing much of anything…[now] I’ve been walking every day, plus going to the gym three times a week.” (TC) • “I do more [now]…I’m riding the bike on a daily basis…I start off doing my Tai Chi…loosening my body and my breathing…then I get on my bike. That’s what they taught me…get on the bike and start riding and…meditating.” (TC) • “I am walking a lot now…I was doing 4000 steps a day, now I’m up to 10,000 steps a day.” (MBB) **Engagement in Other Healthy Behaviors** • "I’m not a very good patient. I wasn’t taking [my inhalers] the way I should be. . .I was kind of careless because I didn’t really want to think that I had COPD…But now I’m more aware and make sure I have them with me at all times” (TC) • " I couldn’t deep breathe very well…When I was smoking it was just more difficult… cause the cigarette smoke was on my hand…I’ve always hated the smell. But when I was into my breathing and connecting with the Tai Chi, it would stand out almost like rotten fish. And I’m like, hmm. Yeah…you can’t do this and smoke." (TC) • "I’ve been losing weight easier. . .I’m more conscious of what I’m eating…now when I sit down for a meal, I realize—wait a minute, what’s this going to do if I take too much or say I have too much salt… how’s it going to affect me? It’s making me think now…it’s helped me with my diet." (MBB)

## Box 3. Representative quotes related to behavior-related neurocognitive/psychological concepts

**Table:** 

**Internal Locus of Control for Disease Management** **Gaining a New Tool or Resource** • “There’s days I didn’t feel like going to gym, days I didn’t feel like doing nothing but stay in my pajamas in the house. But in my mind I says, I gotta do this, nobody can do this for me but me…these are things that I have to do.” (TC) • “[I]t’s made me appreciate my body more and trying to figure out how I can fix it. … I can go ahead and succumb to the arthritic changes and the pain and become a dope fiend–or I can find ways to help myself. I’m more into trying to find some healthy things to do.” (TC) • “[Tai Chi] can make you…believe in yourself… (it) brings more resources, you can do better than you think you can.” (TC) • “It’s given me tremendous strategies to use to deal with COPD, bottom line: I’m in control…If I’m getting out of breath—I have strategies.” (TC) • “When I get in trouble at home [I can use] the tools that they were given to me. [T]here are things that you put a limit on yourself, because in your mind you think you can’t do it, but if you can change that mindset than your body can go along with your mindset…You just learn to adapt…now you have these tools” (MBB) **Self-Efficacy for Symptom Management, Managing Anxiety and Breathlessness, and for Physical Activity** • "I can actually get up the hill by my house sometimes without having to stop now. Yesterday I made two trips to Shaw’s. That would’ve been out of range before. But I watched the DVD and done some Tai Chi, I was like, okay I can do this. It definitely gives me a better feeling about myself." (TC) • “It does make me more aware of either trying not to get myself into a situation where I’m going to have a problem, kind of heading it off, you know. . .or looking at stairs and saying, okay you can do this, you just have to do it with a different pace, and a different breathing exercise. . .trying to apply some of them.” (MBB) • “I had one respiratory infection, and I knew I was going to jump on that right away and not have an exacerbation…and it worked. I was forceful and proactive, in the treatment I wanted.t.” (TC) • “I’m less afraid to attempt to do certain physical activities…since I started the Tai Chi. Because I know…I can recover my breath if I get short winded, and not panic, and that’s one of the things that I know the Tai Chi has taught me.” (TC) • “When you get emotional, you lose that oxygen, you get stressed out…So now…in a stressful situation, I can back up, take the whole scenario and go from there…when you can control your breathing…your nerves will quiet down and you’ll get more oxygen and you won’t [have] anxiety all the time.” (MBB) • “I enjoy walking now…before, I didn’t… I said, damn it I have to go up to this store, and get this, I used to dread it, but now I don’t, because I know how to breathe to get there, and I get there with relaxation.” (MBB) **Acceptance of Disease** • “I’ve always lived with disease. But I’m not the disease.” (TC) • “I realized what my restrictions are and what they aren’t, and I try to maximize what I can… I feel pretty comfortable with myself now.” (TC) • “This disease must be your friend…I’m not going to get rid of it, you know. But I have to be a friend to it and treat it well…” (MBB) **Intention to Continue Self Care** • “..That’s what makes you want to keep practicing [Tai Chi], because, it does help you …I’m aging, I want a better quality of life, I want to take care of myself as long as I can.” (TC) • “Even if it’s just a little part time thing for me, I could open up a little Tai Chi studio somewhere…That’s one of the things that’s kind of kicked up in my mind from the whole study. It’s like ooh I can actually do this, cause it’s not like a job. It’s actually fun and beneficial…[Some]one asked me, “What you’ve been doing? You look different.” I talked to him about the Tai Chi and he’s like, “Oh is that the stuff old people do in the park?” I’m like well yeah, it’s the same thing…So I hope to be one of the guys in the park here this summer or spring.” (TC) • “I’m gonna do it [Tai Chi] the rest of my life. It’s like a life practice.” (TC)

## Box 4. Representative quotes related to physical function

**Table:** 

**Improved Physical Functioning, Reduced Dyspnea, and Reduced Need for Rescue Medications** • “I see changes in my body, doing the Tai Chi…I’d ride [my bike] father and longer…it just gradually climbed, just kept going further and further.” (TC) • "When I feel [shortness of breath] coming on, I’ll slow my pace down and put my mind in a better place and usually I don’t get the tension as much in the chest…I’m going to work through it. I’m not as apt to reach for the inhaler." (TC) • “This is very important to me. . .it used to take me before I started classes an hour before I could even leave the house because I got to put on my shoes, you’d be surprised how much air that usually takes to put on my shoes. . .it is the biggest effort for me…My wife she loves to go to the grocery store … but somebody gotta take them bags upstairs, most likely it’s me… it used to be a problem, I used to dread it, because there’s nobody there but us. . . it’s 8, 9, 10 bags every time she goes shopping, and it’s all gotta get up there, and it’s me that do it. . .But it’s no problem now.” (MBB) • “[Before] I couldn’t. . .make it up a couple of flights of stairs, by the end I was making it up that flight of stairs. And not only was I making it up that flight of stairs, but I was also talking and walking up that flight of stairs.” (MBB) • “I think I cut the lawn, down on the Cape, three times last year…Because the push lawn mower. I couldn’t push it, I’d get out of air. The past 12 weeks, I’ve mowed the lawn every week. I don’t stop halfway through, I don’t stop three quarters of the way through, I start the lawnmower, and I don’t shut it off until I’m all through mowing. I finished the lawn. . .I was still not out of air.” (MBB)

## Box 5. Representative quotes related to psychological wellbeing

**Table:** 

**Emotional Regulation** • “It actually helps, big time… with PTSD it’s easy for us to go from zero to a hundred miles an hour…I can feel it building so then [I can] go into my breathing…I could put a baffle on things…Instead of letting it escalate all the way.” (TC) • “When my granddaughter was first born, I was very stressed–because she was only a pound and a half–and worrying about her. I was able to pick the phone up, take that deep breath, gather my thoughts, and talk to [my daughter]….I learned to control my emotions, and gather information without stressing…” (MBB) **Relaxation and Less Emotional Distress** • " There’s a whole lot of things that are really stressful, like health insurance,…because I’m on a fixed income. . .But, you know, when I did the exercises at home, I did feel a lot less stressed out…I learned how to relax more than I ever could." (TC) • “With all the stress in my life, it was the two days a week that I actually could relax… not take life for granted and be more aware of what’s going on and being able to relax and enjoy it." (MBB) • “I didn’t beat cancer…[just] to die of stress…to me, this type of breathing is a de-stresser.” (MBB) • "I have a lot of things going on in my life. I have rental property and trying to rent apartments and evicting people and maintain bookkeeping processes, and this [the study] has helped me to get some distance.…and let go of things that are really heavy." (MBB)

## Box 6. Representative quotes related to social support and social function

**Table:** 

• “There were different people at different stages and I could see where, okay…if I just let the eating get out of control…Looking across the circle at him, that could be me and I don’t want that.​” (TC) • “[I]t brought my family a little bit closer together. Instead of dad being all curled up in pain, dad is doing these movements with us. . .it’s kind of giving us an added family activity.” (TC) • “I loved it because, my fears are not only my fears, I’m not the [only] crazy woman that says, oh my god, I’m afraid that if I get into an elevator and it is stopped on a floor and is broken, what am I going to do. . .” (MBB) • “I enjoy my grandchildren more because now I’ve got the energy to go far with them, fool with them, walk with them, which I wasn’t doing before….[I]t’s helped me with my wife. We’re getting along a lot better…[previously] I’d just jump and lash out…and now I can reevaluate. . .” (MBB) • “[I]t was a learning experience from everybody…you see a lot of people coping and you’re like, “You’re crazy. I’d never do it that way.”…but at the same time I’ve learned to maybe not pull the trigger as quickly… people who deal with it they have knowledge to share.” (EDU)

### Overall awareness and understanding

Participants expressed being more conscious of, thinking more often about, or having an improved understanding of themselves, their body, their disease, and the mind-body connection. In the TC and MBB intervention groups a majority of participants expressed increased awareness Of Self, a theme which including expressing attention to their body, breathing patterns, movement and posture, and the relationship of these to other symptoms.

Across all three groups participants expressed a new or greater understanding Of Disease. This included an improved understanding basic respiratory physiology and function, the effects of COPD on the lungs, and the disease’s long-term prognosis.

Not surprisingly, only those patients in the TC and MBB intervention groups spoke about the mind-body connection and how it might help COPD symptoms. Both the TC and MBB groups commonly expressed this as an understanding of the integration of mind and body, that this was an important principle of the mind-body interventions taught that is different from other exercise, and that mental health and emotions are linked to and can impact physical well-being.

### Specific self-care knowledge, skills, and behaviors

Across all groups participants commonly discussed how the interventions influenced their knowledge, beliefs, and behaviors around self-care management. In particular, knowledge of breathing techniques and utilization and integration of self-care skills into daily life was more often reported in the intervention groups than the EDU control. Not surprisingly, those in MBB mentioned breathing most often, followed by TC. Another commonly mentioned disease management or self-care strategy was learning how to pace oneself to increase stamina over time.

Beyond knowledge of various self-care strategies, participants discussed specific situations in their daily lives where they were able to utilize behavioral skills learned in class, call upon these skills as needed, and began to incorporate them into their everyday lives. This theme was more prevalent in the two intervention groups.

Participants in all groups mentioned doing physical activities, such as walking, more often than previously. Some participants, primarily in TC and MBB, discussed increased awareness and engagement in healthy behaviors such as smoking less, eating healthier, and improved medication compliance.

### Behavior-related neurocognitive/psychological concepts

Within this domain, locus of control and self-efficacy were common themes that notably occurred more often among TC and MBB participants. Locus of control is the degree in which one perceives control over the outcome of events in one’s life, as opposed to external forces beyond one’s control [42]. Participants in the TC and MBB groups expressed having an internal locus of control for management of their COPD, including control over health-related outcomes. Participants often used similar language in describing increased locus of control and this centered around gaining a new “tool,” they could use to manage symptoms.

Related to locus of control, patients in TC and MBB also expressed increased self-efficacy, or self-confidence or belief in one’s capabilities for specific tasks. We noted mentions of self-efficacy specifically around symptom management, managing anxiety and breathlessness, and engaging in physical activity. In particular, those in TC and MBB mentioned confidence in intervening on anxiety should they feel shortness of breath so that further anxiety/breathlessness did not escalate and further worsen symptoms. Self-efficacy for physical activity was also mentioned slightly more than other groups.

Patients also shared a range of themes related to acceptance of their disease and the limitations or challenges it created, with such expressions occurring most often in TC. Patients often shared that they felt such acceptance helped them adapt, or let go of negative feelings about having COPD.

Another theme that was most prevalent among TC participants was explicit expression of intention to continue physical activity or other management of their disease. Many statements expressed the intention to continue with Tai Chi.

### Physical function

Patients in the TC and MBB intervention groups shared a variety of perceived improvements in their physical wellbeing, especially ease in performing specific activities, more energy, improved breathing, and a reduced need for rescue medications to manage their symptoms.

### Psychological wellbeing

Participants in both intervention groups reported a range of emotional improvements as a result of the study. One prominent theme was characterized by emotion regulation, and being less emotionally reactive or volatile, and more able to manage strong emotions in perspective. Participants provided specific examples of stressful events they felt they had handled more successfully than they might have in the past.

Both intervention groups also reported experiencing less emotional distress—including feelings of stress, sadness, and anxiety, and an improved outlook on the future. Expressions of appreciation for oneself, for others, and for nature, emerged more often in TC. Increased relaxation was mentioned by a significant majority of intervention participants. Intervention participants spoke of relaxation both during and immediately after classes, and of a general sense of greater relaxation in their lives.

### Social support and social function

Patients shared a variety of social benefits from their participation, both during the classes themselves and more broadly. These themes were seen across all three groups. Participants mentioned a benefit from seeing others with the same disease, either as motivation to be more active in their self-care, or because it let them see the way others coped. Participants also expressed the value of sharing a difficult experience with their classmates or experiencing improved social engagement outside of class. Those in all groups, especially EDU, reported having shared knowledge and information about COPD together with their classmates. Feelings of increased social support were primarily connected to time spent in the classes. However, some patients mentioned feeling more socially engaged outside of class, or more able to fulfill important social and familial roles, such as husband or grandparent, often tied to positive changes in other areas, such as increased energy or improved emotional regulation.

## Discussion

The aim of the current study was to qualitatively assess the impact of Tai Chi, mind-body breathing, and an education control in a pilot RCT among individuals with COPD. Several important domains emerged, including 1) overall awareness and understanding of oneself and disease, 2) self-care knowledge, skills, and behaviors, 3) behavior-related neurocognitive concepts, 4) physical function, 5) psychological well-being, and 6) social support and function. Participants in both the TC and MBB interventions, but not the education control group, endorsed many themes within each domain at similar rates, including improved internal locus of control and self-efficacy towards managing anxiety and dyspnea, greater emotion regulation and decreased reactivity, less emotional distress, improved physical function, and valuing social interactions within the program and in their personal network. However, compared to the MBB group, more participants in the TC group reported greater intentions to engage in self-care behaviors, particularly physical activity, and greater self-efficacy for physical activity. Those in education only endorsed increased knowledge and management of disease. Taken together, these findings highlight 1) the impact of mind-body interventions on key health behavior change processes, and 2) the need to better understand the effects of different mind-body interventions for COPD, including the potential added benefits of integrating physical activity.

Recently, the Science of Behavior Change, a NIH Common Fund, identified three higher-order mechanistic pathways for behavior change, including self-regulation, stress reactivity/resilience, and interpersonal/social processes [25, 43]. These themes emerged in both the TC and MBB groups, with some important distinctions, but not the education group. For example, intention, or specific plans about how behavior change will occur, has been identified as a necessary self-regulatory process in health behavior change models, such as the theory of planned behavior and the transtheoretical model [16, 44, 45]. In our study, half of the participants in TC, compared to few in the MBB group, endorsed an intention to continue self-care behaviors, particularly physical activity. This finding supports a hypothesis that a multimodal intervention, which combines mind-body breathing and physical movement (i.e., Tai Chi), may offer advantages over mind-body breathing alone (i.e., a single component of Tai Chi) in regards to increasing physical activity and other self-care behaviors.

Self-efficacy is another important self-regulatory health behavior change process that was frequently endorsed by both the TC and MBB groups. Intervention participants expressed similar improvements for self-efficacy related to symptom and anxiety management. However, more participants in the TC group endorsed increased self-efficacy for engaging in physical activity, specifically. Given the similar levels of improvement between the intervention groups for self-efficacy in other areas, it seems possible that the integration of meditation, breathing, and physical activity, which is central to the practice of Tai Chi, may increase confidence in one’s ability to engage in physical activity. These findings support prior studies of Tai Chi in other chronic cardiopulmonary conditions that suggest a favorable impact on exercise self-efficacy [46, 47].

Emotion regulation, or one’s ability to modulate affective states, has also been implicated as a self-regulatory process key to health behavior change [48]. Strikingly, more than half of participants in the TC and MBB groups reported improvements in emotion regulation, while this theme was entirely absent among the EDU group. At least one prior study in COPD found that Tai Chi may have a positive effect on emotional regulation [12]. Together, these findings suggest that both TC and MBB alone enhance one’s ability to modulate difficult emotional states, which may produce downstream improvements in healthy behaviors and psychosocial functioning. More rigorous research is needed to examine whether MBB alone is sufficient to improve emotion regulation, or whether multimodal mind-body interventions, such as Tai Chi, add benefits above and beyond this specific component.

Acceptance has been increasingly recognized as a potential health behavior change process [49]. Around a third of TC and MBB participants expressed acceptance of their disease or limitations, with many explicitly linking this acceptance to an improved ability to engage in behaviors to better manage their COPD. Limited prior research in this area suggests that acceptance of COPD is associated with improved quality of life [50] and fewer depression symptoms [51]. Additionally, acceptance has been correlated with improved medication adherence [52], greater self-management of exacerbations [53], and positive behavioral changes during pulmonary rehabilitation programs [54]. Overall, these findings suggest that acceptance may be an important factor in behavior change for COPD patients and that Tai Chi and MBB may be well-suited for targeting this process.

Results from the current study suggest that more rigorous research is needed to better understand the effects of different mind-body interventions for COPD, including the potential added benefits of integrating physical activity and the implications for promoting positive behaviors. Indeed, participants in the MBB and TC groups equally endorsed most qualitative themes related to emotional and physical functioning. Both groups also reported an increase in self and interoceptive body awareness, which is one important aspect of mind-body therapies [27, 55]. However, one major distinction between the two groups was that those in the TC group (which integrates MBB with physical activity) more frequently endorsed themes related to self-efficacy for physical activity and intentions to engage in physical activity. Indeed, prior literature has suggested that Tai Chi may promote physical activity and increase exercise capacity in diverse cardiopulmonary populations [56, 57]. Our current findings support the hypothesis that body/breath awareness and mindful physical activity may interact in multimodal Tai Chi interventions to provide benefits above and beyond mind-body interventions with no physical activity component (e.g., MBB) [27, 58, 59]. Quantitative comparison between the TC and Edu group, published in full elsewhere [41], provide preliminary support for improved self-efficacy, especially towards positive self-care behaviors and physical activity, and decreased psychological distress among intervention participants.

While traditional pulmonary rehabilitation programs for COPD do improve patient’s physical function [60, 61], self-efficacy [54], and quality of life [61], these benefits tend to diminish within six months of completing the program [60, 62]. Supervised and home-based exercise programs designed to support long-term maintenance of physical activity and prolong benefits have had mixed results [63–65]. These qualitative results support the consideration of mind-body exercises in in the development of novel programs to address physical activity maintenance in this population.

Our findings also highlight the value of assessing patient-centered outcomes consistent with biopsychosocial model among patients with COPD. For example, while critically important diagnostically, FEV_1_ often fails to capture the breadth of the disease’s impact on patients and therefore correlates only modestly with health status and satisfaction [66]. Patient-centered outcomes such as self-efficacy towards symptom management and self-care, the ability to address anxiety and breathlessness, or overall psychological well-being were significantly meaningful to patients with COPD and may be equally important to care as results of radiographic imaging or pulmonary function tests.

There are several limitations to this study that are important to acknowledge. First, the interviewer who conducted the qualitative interviews was a member of the study team, and participants may have offered more positive statements to please the investigators. Second, we recognize theoretical overlap in some of the themes and sub-themes such that coding decisions may be subjective. Third, due to the open-ended nature of the interview, theme separation or number of endorsements of a single theme within an individual’s interview was not always clear. To reduce bias in these areas, we randomly selected transcripts for analysis, had independent coding and theme extraction by two authors, and used triangulation where a third arbiter was used in case of any discrepancies. Our descriptive comparisons and conclusions are exploratory and should be replicated or confirmed using quantitative methods.

Despite these limitations, these findings generate valuable hypotheses regarding pathways by which mind-body therapies may impact behavior change for patients with COPD. These findings also suggest that there may be specific advantages associated with a multimodal Tai Chi intervention (i.e., that combines MBB and physical movement), particularly for promoting physical activity engagement and this should be further studied.

## Supporting information

S1 Appendix Parent study consort diagram. (TIF)

S2 Appendix Semi-structured qualitative interview guide. (DOCX)
