# Supplementary figures and images for: The impact of Tai Chi and mind-body breathing in COPD: Insights from a qualitative sub-study of a randomized controlled trial
Source: PLoS One. 2021 Apr 8;16(4):e0249263. doi: 10.1371/journal.pone.0249263 (PMC8031883; doi:10.1371/journal.pone.0249263)

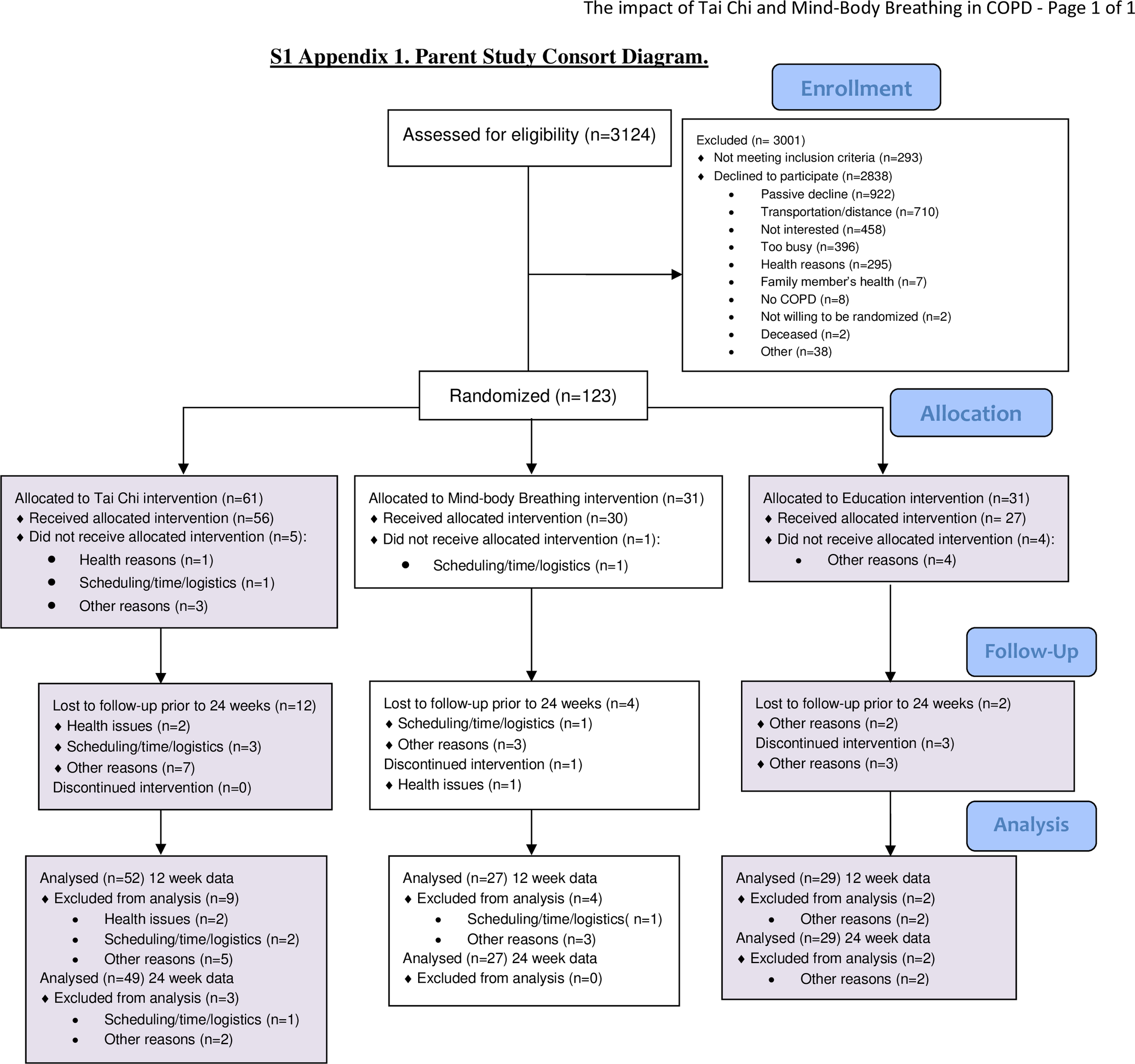

Supplement: S1 Appendix — (TIF) [file pone.0249263.s001.tif]
