# Supplementary material for: The impact of Tai Chi and mind-body breathing in COPD: Insights from a qualitative sub-study of a randomized controlled trial
Source: PLoS One. 2021 Apr 8;16(4):e0249263. doi: 10.1371/journal.pone.0249263 (PMC8031883; doi:10.1371/journal.pone.0249263)
Supplement: S2 Appendix — (DOCX) [file pone.0249263.s002.docx]

**S2 Appendix. Semi-Structured Qualitative Interview Guide.**

**Introduction**

- Thank you for agreeing to participate in this interview. I want to let you know that there are no right or wrong answers to these questions. You can decline to answer any of these questions. You will remain anonymous on this recording. We are interested in your experiences with the program and with the tai chi/MBB/education. Any thoughts, feelings, experiences you can share with us will be very helpful.

**Illness/Medical Condition/Life**

*Allow up to 5 minutes*

- To start, let me ask you to think about your pulmonary condition. Tell me the story of your COPD.
  - How has your COPD impacted your life?

**The Program**

*Allow about 5 minutes*

The next few questions are about being a participant in a research study , which includes testing visits and follow-up. You may also talk about the classes themselves but there will be opportunities later to talk specifically about your (TC, MBB, Education) experience.

- Tell me about your experience in the study during the last 12 weeks. What stood out for you?
- Has participation in the study changed your life in any way? If so, how?

*Positive aspects*

- Was this study helpful? How?
  - What have you learned?
  - What do you feel has been the most important aspect of what you may have learned?

Negative aspects

- What was challenging or difficult for you in the study?
- What would you change?
- Did you experience anything that concerned you?

Group

- What was it like to be in a group with others with the same condition?

**Specific Questions About Tai Chi/MBB** (for Tai Chi/MBB participants only)

*Leave about 30 minutes for the remaining questions*

- For the next group of questions, we’d like you to think specifically about the TC/MBB practice. We are interested in understanding if you have experienced any changes in relation to your COPD or other aspects of your life as a result of your participation in the TC/MBB.
- Do you think this practice affected you? In what way? How?

Physical function

- Have there been any changes in your physical functioning?
- How about with respect to everyday activities like walking, chores, breathing, did the TCC/MBB affect these in any way?
  - In this class, we tried to simulate some activities of your daily life, like walking, and speaking on the phone, among other things, while still incorporating the breathing techniques learned in class. What did you think of these exercises and were they helpful or not?
- Have there been any changes in your body? How did those changes feel?
  - e.g., symptoms such as breathlessness, fatigue, aches, pains
- Did the TC/MBB change the way you use your inhalers, nebulizers, oxygen, any over the counter medications?

Mental function

- Have there been any changes in your mental functioning?
  - e.g., memory, executive function, mental tasks, psychological health, mood
- How about with respect to managing stress and other demands, did the TC/MBB affect this in any way?

Social Function

- Have there been any changes in your social life, with respect to relationships with family or friends? What about changes in how you interact with family members, friends, or people in general?

Illness perception

- As a result of your participation in TC/MBB, did your understanding of your condition change?
  - Did it change the way you manage your condition?
  - Did it change your approach to your condition?
  - Did it change the way you think about your condition?
  - Did it change your outlook? Your thoughts about what will happen in the future?

Intervention practice

- Can you tell me how you feel TC/MBB is similar to other forms of exercise?
- Can you tell me how you feel TC/MBB is different from other forms of exercise?
- Have you experienced barriers that make it difficult to practice tai chi/MBB?
  - (both at practice outside of class, and tai chi practice in general)
- Imagine yourself in the place where you most often practice tai chi/MBB, can you describe a typical practice experience?
  - (what is your set-up like, time, place?)
- When you practiced, how did you feel? What was it like for you?
  - Did you become more aware of your body?
  - Did you become more aware of your environment?
  - Did you experience changes in your energy?

**Expectations/Beliefs**

- Was the tai chi/MBB exercise class what you had expected? If so, how? If not, why not?
- How do you think tai chi/MBB works? How did you get that idea?
- Do you believe that Tai chi/MBB practices can affect your mental health (for example, your mood)?
- Do you believe that Tai chi/MBB practices can affect your physical health (for example, your lung function)?
- Finally, is there anything else you would like to tell me about your experiences in this program?

**End of Interview**

- Thank you very much for your time and participation in this study. We are very grateful.

**Developed for the BEAM Study (NIH R01AT005436; PI Yeh, Gloria);**

**Beth Israel Deaconess Medical Center, Harvard Medical School**
